# A Recombinant La Sota Vaccine Strain Expressing Multiple Epitopes of Infectious Bronchitis Virus (IBV) Protects Specific Pathogen-Free (SPF) Chickens against IBV and NDV Challenges

**DOI:** 10.3390/vaccines7040170

**Published:** 2019-11-01

**Authors:** Lei Tan, Guoyuan Wen, Xusheng Qiu, Yanmei Yuan, Chunchun Meng, Yingjie Sun, Ying Liao, Cuiping Song, Weiwei Liu, Yonghong Shi, Huabin Shao, Chan Ding

**Affiliations:** 1 Department of Avian Diseases, Shanghai Veterinary Research Institute, Chinese Academy of Agricultural Sciences, Shanghai 200241, China; xsqiu1981@shvri.ac.cn (X.Q.); yuanyanmei@shvri.ac.cn (Y.Y.); mengcc@shvri.ac.cn (C.M.); sunyingjie@shvri.ac.cn (Y.S.); liaoying@shvri.ac.cn (Y.L.); scp@shvri.ac.cn (C.S.); liuweiwei@shvri.ac.cn (W.L.); shiyonghong@shvri.ac.cn (Y.S.); 2 Institute of Animal Husbandry and Veterinary Sciences, Hubei Academy of Agricultural Sciences, Wuhan 430070, China; wgy_524@163.com; 3 Jiangsu Co-innovation Center for Prevention and Control of Important Animal Infectious Diseases and Zoonoses, Yangzhou 225009, China

**Keywords:** Newcastle disease virus vector, infectious bronchitis virus, multi-epitope vaccine, challenge, cross-protection

## Abstract

Infectious bronchitis (IB) and Newcastle disease (ND) are two major infectious diseases that are a threat to the domestic poultry industry. In this study, we successfully generated a recombinant LaSota candidate vaccine strain, rNDV-IBV-T/B, which expresses a short, synthetic, previously identified IBV S1 multi-epitope cassette using the reverse genetic system. The recombinant virus was propagated in nine-day-old embryonated chicken eggs for 20 passages and genetic stability was confirmed by whole genome DNA sequencing. The recombinant virus had a hemagglutination (HA) titer of 2^10^, mean death time (MDT) of 118 hours, and intracerebral pathogenicity index (ICPI) of 0.05. None of these were significantly different from the parental Newcastle disease virus (NDV) LaSota strain (*p* > 0.05). Vaccination of white leghorn chickens at one day of age with 10^6^ EID_50_ rNDV-IBV-T/B provided 90% protection against virulent IBV M41 challenge at three weeks of age, which was significantly higher than the protection of the control group vaccinated with phosphate-buffered saline (PBS) (*p* < 0.05). The ciliostasis scores of rNDV-IBV-T/B-vaccinated and LaSota-vaccinated groups were 4.2 and 37.6, respectively, which indicated that rNDV-IBV-T/B vaccination reduced the pathogenicity of IBV toward the trachea. Furthermore, real-time RT-PCR assay showed that the rNDV-IBV-T/B vaccination resulted in low levels of viral load (647.80 ± 49.65 RNA copies) in the trachea four days post-challenge, which is significantly lower than groups vaccinated with PBS (8591.25 ± 311.10 RNA copies) or LaSota (7742.60 ± 298.50 RNA copies) (*p* < 0.05). Meanwhile, the same dose of rNDV-IBV-T/B vaccination provided complete protection against velogenic NDV F48E9 challenge. These results demonstrate that the rNDV-IBV-T/B strain is a promising vaccine candidate to control both IB and ND simultaneously. Furthermore, epitope-based live vector vaccines provide an alternative strategy for the development of cost-effective and, broadly, cross-protective vaccines.

## 1. Introduction

Avian infectious bronchitis (IB) is an acute and highly contagious viral respiratory disease of chickens. IB occurs worldwide and causes substantial economic losses in the poultry industry [1,2,3,4,5]. IB is caused by the infectious bronchitis virus (IBV) which belongs to the order *Nidovirales*, family *Coronaviridae*, genus *Gammacoronavirus*. IBV causes upper respiratory illness, nephritic syndrome, and a drop in egg production in layer chickens [1]. The mortality rate can be high in young chickens, especially with other secondary complications such as viral and bacterial infections [6,7,8,9,10].

Currently, novel serotype and variant IBV have emerged due to rapid mutation rates, viral recombination, and host selection pressure, which have resulted in IB prevention becoming a global challenge [11,12]. Vaccination is the best method to control the disease, and the current widely used commercial IBV vaccines are live-attenuated or inactivated viruses with adjuvants derived from classical or variant serotypes [13], however, live-attenuated IBV vaccine strains have some limitations, including genetic instability, reversion to virulence, and recombination between vaccine and wild-type viruses [14]. IBV-inactivated vaccines also face some defects such as lack of ability to elicit strong cellular immune responses and lack of cross-immunoprotective effects. Furthermore, chicken flocks suffer co-infection with IBV and avian orthoavulavirus-1 (AOAV-1), which is a member of the genus *Orthoavulavirus* of the family *Paramyxoviridae*, often referred to as Newcastle disease virus (NDV) [15], which hinders the prevention and control of these diseases [16,17]. These two pathogens remain a vital threat to domestic poultry production [18,19,20,21]. Thus, it would be advantageous to develop a next-generation vaccine that could serve as a bivalent vaccine against IBV and NDV challenge. 

In recent years, epitope-based vaccines have been widely studied for their advantages, including safety, genetic stability, ease of production, and the ability to elicit immune responses against multi serotype pathogens. [22,23]. Thus, they have been broadly applied for a range of viral, bacterial, and parasitic diseases [24,25,26]. The most important factors for designing a multi-epitope vaccine are predicting and screening the functional neutralizing B cell epitopes and species-restricted T cell epitopes [27,28]. IBV spike glycoprotein (S) is proteolytically cleaved into a 92-kDa S1 subunit and a 84-kDa S2 subunit, and these play an important role in viral infections [29]. The S1 subunit possesses a receptor binding domain (RBD) that is primarily responsible for adsorption and entry into host cells. Epitopes of neutralizing antibodies are also found mainly on the S1 subunit [30,31]. Therefore, the S1 protein has an indispensable effect on the protective immune response of IBV. Our previously constructed IBV S1 protein multi-epitope cassette includes sequences encoding neutralizing epitope domains (aa 24–61, aa 132–149, and aa 291–398) of IBV Australian T strain (Genbank AY775779.1) and BF2-restricted CTL epitopes aa 413–421 and aa 517–525 of the QX-like IBV SH1208 strain, which shares an ancestor with the SAIBK strain (Genbank DQ288927.1), and aa 45–52 and aa 413–421 of the Holte strain (Genbank L18988.1). The immune-protection experiment demonstrated that the recombinant DNA vaccine, consisting of this IBV S1 protein multi-epitope cassette, could induce a high level of immune responses and full protection of chickens against a lethal dose of IBV challenge [26,32]. The results indicated that the multi-epitope-based vaccine provides a promising strategy for vaccine development against IBV infection. 

The technology of the reverse genetic system (RGS) for NDV was established in Europe two decades ago, which has been successfully applied to research on pathogenicity, replication regulatory mechanisms, and vaccine vectors [33]. Meanwhile, the LaSota strain has been used as a safe and effective live vaccine that protects against NDV challenge for several decades, because it can elicit both humoral and cell-mediated immune responses, and vaccine production is cost-effective. [34]. Thus, it would be very attractive to rescue a rNDV LaSota strain expressing the immunogenic proteins or functional epitopes of other respiratory viruses, such as the S1 protein of IBV. 

The aim of this study was to develop a live vaccine using recombinant NDV expressing previously identified IBV multi-epitope cassette. The protective efficacy of the recombinant NDV vaccine was evaluated by heterologous IBV and velogenic NDV challenge. The findings could provide an alternative and promising strategy for the development of a cost-effective and extensively immune-protective vaccine for the control of ND and IB. 

## 2. Materials and Methods 

### 2.1. Cells, Viruses, and Ethics Statement

The chicken embryonic fibroblast cell line (DF-1) and baby hamster kidney fibroblasts clone 13 cell line (BHK-21) were obtained from the American Type Culture Collection (ATCC, Manassas, VA). They were grown in Dulbecco’s minimal essential medium (DMEM) containing 10% fetal bovine serum (FBS). Fertile white leghorn SPF embryonated eggs were purchased from the Beijing Merial Vital Laboratory Animal Technology Co., Ltd. The recombinant avirulent NDV LaSota strain was developed previously in our laboratory by a reverse genetics system [35,36]. The velogenic NDV genotype IX strain F48E9 [37] and Massachusetts IBV strain M41 obtained from the China Institute of Veterinary Drug Control (Beijing, China) were stored in our laboratory at –70 °C. The 50% embryo infectious dose (EID_50_) of a virus in harvested allantoic fluid was calculated by the Reed and Muench mathematical technique [38]. All experiments involved standard procedures with the formal approval of the Ethics and Animal Welfare Committee of Shanghai Veterinary Research Institute, China (Approval No. Shvri-po-20180616).

### 2.2. Construction of rNDV Containing an IBV S1 Protein Multi-Epitope Cassette

We employed NDV as virus vector and rescued the recombinant NDV expressing an IBV S1 protein multi-epitope cassette via a reverse genetics system. Briefly, five overlapping cDNA fragments covering the complete genome of the NDV LaSota strain were amplified by RT-PCR using genomic RNA as a template. The five cDNA fragments were subsequently assembled into a modified pBR322 vector under the control of the T7 promotor and followed by a partial Hepatitis D virus (HDV) ribozyme and T7 terminator, resulting in a full-length cDNA clone, named pBR-LS.

The IBV S1 multi-epitope cassette S-T/B, constructed previously, was inserted between P and M genes in the LaSota genome as an additional transcriptional unit [26]. The full-length plasmid clone pBR-LS was linearized at the 5′-NTR of the NDV LS M gene by PCR using PFU Ultra Fusion HS DNA polymerase (Agilent, Santa Clara, CA, USA) and vector-specific primers to include the complete sequence of this clone. The S-T/B fragment fused with gene-end (GE), intergenic (IG), gene-start (GS), and Kozak sequences was amplified by overlapping PCR and ligated with the linearized pBR-LS using an In-fusion PCR clone kit (Clontech, Mountain View, CA, USA). The constructed plasmid, named pNDV-IBV-T/B, was confirmed by sequencing analysis. The NP, P, and L genes of LaSota were amplified from the pBR-LS plasmid, and the PCR products were subcloned into the pVAX1 vector (Thermo Fisher Scientific, Waltham, MA, USA) to obtain helper plasmids named pV-NP, pV-P, and pV-L, respectively. The pNDV-IBV-T/B, pV-NP, pV-P, and pV-L plasmids (ratio of 4:2:1:1) were co-transfected into the replication-deficient vaccinia virus (VV) MVA-T7-infected BHK-21 cells with Lipofectamine™ 3000 reagent (Thermo Fisher Scientific, Waltham, MA, USA). At 6 h post-transfection, cells were washed twice, then cultured in DMEM containing 1% penicillin (10,000 IU)-streptomycin (10,000 μg/mL) and 0.2 μg/mL N-tosyl-L-phenylalanine chloromethyl ketone (TPCK)-trypsin for another 72 h. The viruses were harvested by freeze-thawing for 3 times and serially passaged 20 times (P20) in 10-day-old SPF chicken embryos. 

The recombinant LaSota virus, named rNDV-IBV-T/B, P1-P20 passages were detected by reverse transcription polymerase chain reaction (RT-PCR) and all the passages rNDV-IBV-T/B whole genome were analyzed by DNA sequencing (Shanghai Sangon Biotect Co., Ltd., Shanghai, China). Briefly, RNA was extracted from allantoic fluid of rNDV-IBV-T/B-infected chick embryos (QIAGEN, Hilden, German) and reverse transcribed to cDNA with AMV Reverse Transcriptase (Promega, Madison, WI, USA) and random primers (Thermo Fisher Scientific, Waltham, MA, USA) at 42 °C for 30 min. To amplify the target gene IBV multi-epitope cassette, RT-PCR was performed using 2 µL of rNDV-IBV-T/B cDNA, 10 µmol each of the primers (F-5’ GCTAGCGCCACCATGGGAAATTACGTTT-3’, R-5’- CTCGAGTTACGGTTGACTACCAGTAGCG-3’), 10 µL PCR Master Mix (2X) (Thermo Fisher Scientific, Waltham, MA, USA) in a total volume of 20 µL. PCR cycling procedure began with an initial hold at 95 °C for 10 min, followed by 30 cycles containing 94 °C for 20 s, 53 °C for 30 s and 72 °C for 20 s and a final step of 72 °C for 10 min. RT-PCR amplification product was examined by agarose-gel electrophoresis.

### 2.3. Virus Titration and Pathogenicity Assessment

The infectivity of the recombinant virus rNDV-IBV-T/B and parental LaSota strain were characterized using the standard hemagglutination test (HA) and the 50% tissue infectious dose (TCID_50_) assay on DF-1 cells in 96-well plates, and the 50% egg infective dose (EID_50_) assay in nine-day-old SPF chicken embryos, according to *A Basic Laboratory Manual for the Small-Scale Production and Testing of I-2 Newcastle Disease Vaccine* [39]. Virulence of rNDV-IBV-T/B was assessed according to the standard mean death time (MDT) and intracerebral pathogenicity index (ICPI) tests based on the OIE method (http://www.oie.int/en/standard-setting/terrestrial-manual).

### 2.4. Western Blot Analysis

The antigenicity of the multi-epitope cassette chimera of rNDV-IBV-T/B was analyzed by Western blotting. Briefly, 90% confluent monolayers of DF1 cells were infected at a multiplicity of infection (MOI) of 1 with P1 and P20 passages of rNDV-IBV-T/B and the parental virus LaSota strain. DF1 cells were harvested 36 h post-infection, lysed, and proteins were separated on a 10% SDS polyacrylamide gel (SDS-PAGE) under denaturing conditions. After electrophoresis, proteins were transferred onto polyvinylidene fluoride (PVDF) filter membrane. Then, the membrane was incubated in 5% skim milk with PBST buffer (PBS, pH 7.2 containing 0.05% Tween 20) for 1 h at room temperature, followed by incubation with a 1:5000 dilution of rabbit anti-IBV strain polyclonal antibody or mouse monoclonal NDV NP protein antibody in PBST buffer for 1 h at 37 °C. After triplicate washes with PBST buffer, the PVDF membrane was incubated with an HRP-conjugated goat anti-rabbit or rabbit anti-mouse secondary antibody. The protein was visualized using the Pierce ECL substrate kit (Thermo Fisher Scientific, Waltham, MA, USA) and imaged using a Tanon 5200 automatic chemiluminescence image analysis system (Tanon, Shanghai, China).

### 2.5. Recombinant NDV Purification and Detection by Transmission Electron Microscopy (TEM)

The rNDV-IBV-T/B was propagated in nine-day-old SPF chicken embryos and purified by sucrose gradient centrifugation according to a previous report [40]. Briefly, the harvested allantoic fluid was centrifuged at 1500× *g* for 20 min to remove the debris and collect the supernatant. Four milliliters of rNDV-IBV-T/B were layered on top of a step gradient of 60%, 50%, 40%, and 25% w/v sucrose prepared in Milli-Q water. Ultracentrifugation was carried out at 120,000 ×g in a SW41 Ti rotor (Beckman Coulter, CA) for 4 h, at 4 °C. The virus-containing band between the 40% and 50% sucrose layers was collected and 5 μl of rNDV-IBV-T/B virus was placed onto the shiny side of an EM grid and then adsorbed for ~3 min. The grid was stained immediately after virus adsorption 3 times with 2% uranyl acetate for 45 s. After negative staining, observation was by an 80 kV FEI Tecnai Spirit TEM T12 (Thermo Fisher) at 98,000× magnification.

### 2.6. The Protective Efficacy of rNDV-IBV-T/B against Velogenic NDV and IBV Challenge

To analyze the protective efficacy of the rNDV-IBV-T/B vaccine against NDV and IBV infection, a total of 90 one-day-old SPF white leghorn chicks were randomly divided into six groups and housed in negative pressure isolators with sterile water and enough feed. Groups A and B were administered with rNDV-IBV-T/B at dose of 10^6^ EID_50_ per chicken, and groups C and D were inoculated with LaSota at the same dose via the oculonasal route. The control groups E and F were inoculated with PBS by the same route.

Three weeks post-vaccination, groups A and C, and control group E were challenged with 10^6^ EID_50_ of the IBV M41 strain via the oculonasal route, and groups B and D, and control group F were challenged with 10^6^ EID_50_ of the velogenic NDV strain F48E9 by the same route. The lethal dose of the challenge virus had been determined by an experimental chicken infection study. Five challenged chickens from groups A and C, and control group E were subjected to ciliostasis tests at 4 days post-challenge (dpc). The other chickens of each group were monitored daily for symptoms and mortality for another two weeks. The immune-protection and challenge experiments were conducted in triplicate.

### 2.7. Serological Assays

In order to evaluate the antibody responses elicited by rNDV-IBV-T/B, the vaccinated chickens were bled through the wing veins, and sera were isolated for serological tests 21 days post-immunization. HI assays were conducted in V-bottomed microwell plates according to the standard protocol of the OIE Manual to determine the level of antibody titers mounted against NDV in chickens vaccinated by rNDV-IBV-T/B. [41]. Ten serum samples were randomly isolated from the rNDV-IBV-T/B, LaSota, and PBS immunization groups. All tests were repeated in duplicate. The HI titers were expressed as the reciprocal of log2. Virus neutralization (VN) assays were performed as previously description by [26]. In brief, 0.1-ml aliquots of serum were diluted two-fold with PBS. The diluted serum was mixed with an equal volume of 10^2^ EID_50_ of M41 strain, followed by incubation for 1 h at 37 °C. Neutralizing antibody titer was estimated by inoculating nine-day-old SPF embryonated chicken eggs with the incubated virus-serum mixture via the allantoic cavity route. PBS was used as the negative control. The embryonated eggs were examined daily for 5 days. VN titers were defined as the reciprocal of the highest dilution without any mortality and IBV specific lesions on chicken embryos. All the assays were performed in triplicate and VN titers were expressed as the mean ± SEM.

### 2.8. Efficacy of rNDV-IBV-T/B in Preventing IBV Virion Shedding

To evaluate the efficacy of rNDV-IBV-T/B in preventing shedding of IBV in immunized chickens, tracheal swab samples were randomly taken from five chickens from each group 4 dpc. RNA was extracted using an using a High Pure RNA Isolation Kit (Roche, Mannheim, Germany) according to the manufacturer’s instructions. The primers, probe, and PCR conditions for the assay used to detect IBV RNA using RT-qPCR were previously described in the literature [26,42]. Taqman probe was synthesized by Sangon Biotech (Shanghai, China). Briefly, the IBV RNA was amplified using the forward primer IBV5′GU391 5′-GCTTTTGAGCCTAGCGTT-3′; the reverse primer IBV5′GL533 5′-GCCATGTTGTCACTGTCTATTG-3′, and detected by TaqMan dual-labeled IBV5′G probe (5′-FAM-CACCACCAGAACCTGTCACCTC-BHQ1-3′) with CFX96 real-time PCR detection system (Bio-Rad, Hercules, CA, USA). The cycling program was set up with 95 °C for 3 min followed by 40 cycles of 95 °C for 15 s, 56 °C for 25 s and 72 °C for 20 s. The number of IBV RNA copies for each group was based on a constructed standard curve. The standard deviation values were calculated from triplicate reactions.

### 2.9. Ciliostasis Test

The ciliostasis activity of the tracheal explants was examined on 10 tracheal ring explants per chicken (5 chickens of each group) 5 dpc. The tracheas were carefully removed and examined for ciliary activity within 2 h after collection as described in the referenced papers [43,44]. Briefly, 1 to 2 mm sections from the middle of each trachea were prepared and examined under a low-power microscope, and ciliary activity was scored as follows: 0, denotes all beating cilia; 1, 75% beating; 2, 50% beating; 3, 25% beating; and 4, absence of beating (100% ciliostasis). A maximum possible ciliostasis score of 40 suggested complete ciliostasis (total lack of protection).

### 2.10. Statistical Analysis

GraphPad Prism v5.01 was used for statistical analysis. One-way ANOVA with repeated measures was used to evaluate the effect of the vaccines through HI and VN tests. The survival rates of immune protection experiments were determined by log-rank tests, *p* values < 0.05 were considered statistically significant. The data are expressed as the means ± SEM.

## 3. Results

### 3.1. Construction of Recombinant NDV Expressing IBV S1 Protein Multi-Epitope Vaccine (rNDV-IBV-T/B)

**Figure 1:**
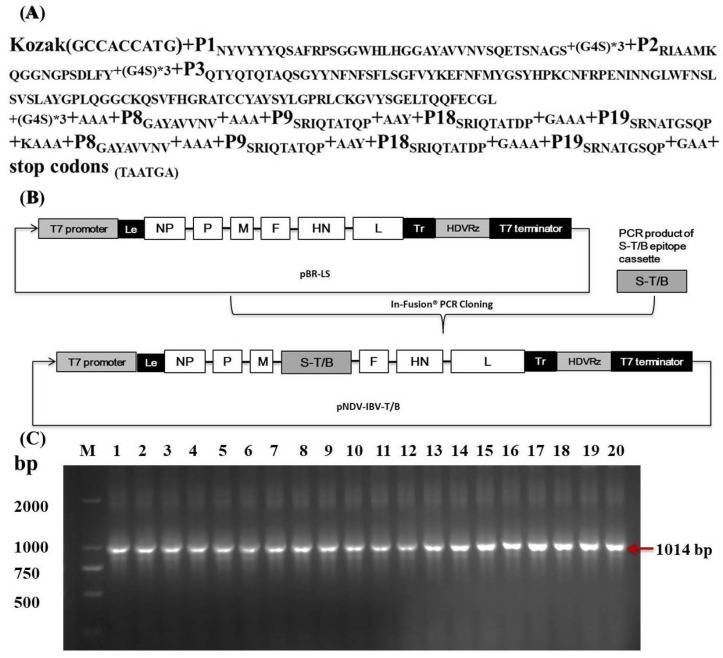
Scheme of rNDV-IBV-T/B construction. (**A**) The open reading frame (ORF) of the IBV S1 T/B multi-epitope cassette gene was amplified from the plasmid pV-S1B+S1T. The cassette contained the Kozak sequence, three neutralizing epitopes (P1–P3), four BF2-restricted T cell epitopes (P8, P9, P18, P19), and was linked with flexible amino acids. (**B**) The multi-epitope cassette ORF was inserted into the pBR-LS vector in the noncoding region downstream of the M gene using the In-Fusion PCR cloning kit, resulting in the pNDV-IBV-T/B clone. The T7 promoter is indicated by a bold black box. HDVRz represents the site of the hepatitis delta virus ribozyme sequence. (**C**) Agarose gel electrophoresis after 20 passages of rNDV-IBV-T/B via RT-PCR; the amplified bands were all identical.

The multi-epitope cassette (Figure 1A) containing the identified S1 protein B cell neutralizing epitopes and BF2-restricted T cell epitopes of IBV was cloned into the full-length cDNA backbone of the LaSota strain between the P and M genes via In-Fusion PCR to obtain the recombinant plasmid pNDV-IBV-T/B (Figure 1B). the pNDV-IBV-T/B was co-transfected with three helper plasmids, pV-NP, pV-P, and pV-L, into MVA-T7-infected BHK cells and then incubated for 72 h at 37 °C and 5% CO_2_. The cell lysates were harvested, filtered, and inoculated into SPF embryonated chicken eggs. The rNDV-IBV-T/B was serially passaged 20 times (P20), and the sequence of the full-length multi-epitope cassette S-T/B was examined by RT-PCR (Figure 1C) and sequencing. The results confirmed that rNDV-IBV-T/B was successfully rescued and genetically stable.

### 3.2. Expression of the S1 Protein Multi-Epitope Cassette of IBV

**Figure 2:**
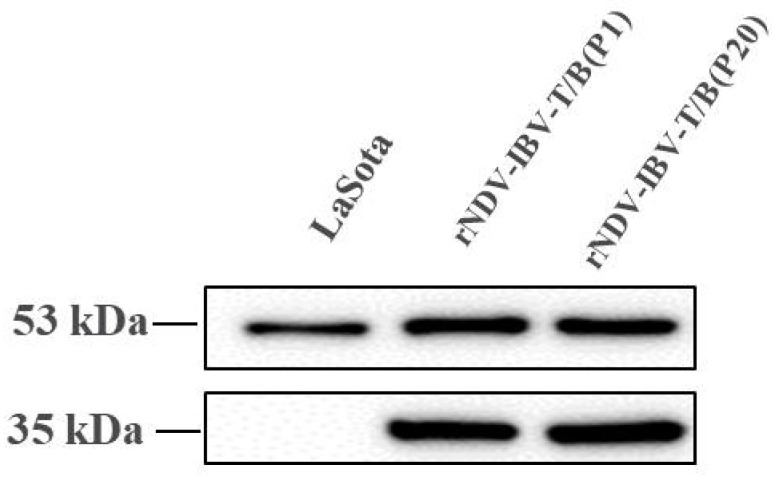
Western blotting of rNDV-IBV-T/B in vitro. DF1 cells were infected with one MOI rNDV-IBV-T/B and the parental NDV LaSota strain. Samples were harvested from DF1 cell lysates 24 h post-infection, and IBV-T/B epitope cassette and NP proteins were detected as approximately 35 kDa and 53 kDa bands, respectively. The LaSota strain was only detected as an NP protein band.

One MOI of two different passages (P1 and P20) of rNDV-IBV-T/B and the parental strain LaSota were used to infect DF1 cells for 24 h, and the cell lysates were harvested and analyzed by Western blot using rabbit polyclonal anti-IBV serum and a mouse monoclonal anti-NDV/NP antibody. The protein expressed from the IBV S1 protein multi-epitope cassette and the NDV NP protein were found at ~35 and 53 kDa bands in P1 and P20 of rNDV-IBV-T/B lysates, however, the parental virus strain LaSota only had the ~53 kDa NP protein band (Figure 2), confirming that rNDV-IBV-T/B successfully expressed the IBV S1 protein multi-epitope cassette in vitro and that the protein was antigenic, as evidenced by its recognition on a Western blot by anti-IBV serum. The expression of the IBV S1 protein multi-epitope cassette, through at least twenty passages of the recombinant NDV, remained stable, indicating genetic stability of rNDV-IBV-T/B.

### 3.3. Biological Characterization of rNDV-IBV-T/B

To determine whether inserting the IBV S1 protein multi-epitope cassette into the NDV LaSota genome affects the biological properties of the recombinant virus, the growth characteristics and pathogenicity after 20 passages of rNDV-IBV-T/B were evaluated through virus titration, MDT, and ICPI tests in vitro and in vivo. The titers of the recombinant virus, determined either in embryonated eggs or in DF-1 cells, were comparable to those of the parental LaSota strain. The EID_50_ titers of the parental virus LaSota and rNDV-IBV-T/B reached 6.5 × 10^8^/ml and 6.7 × 10^8^/mL, respectively. The TCID_50_ titer of rNDV-IBV-T/B (3.7 × 10^7^/mL) was slightly higher than that of LaSota (3.6 × 10^7^/mL) at 48 h post-infection. The HA titers of LaSota and rNDV-IBV-T/B were the same (2^10^). The statistical analysis of EID_50_, TCID_50_, and HA results indicated that there were no significant differences between rNDV-IBV-T/B and the parental LaSota virus (*p* > 0.05). The recombinant NDV remained stable, with no apparent changes in MDT and virus titers after 20^t^ passages in SPF chicken embryonated eggs. Furthermore, rNDV-IBV-T/B has a lower ICPI (0.05) than LaSota (0.10) (Table 1). These results suggest that the presence of the IBV S1 protein multi-epitope cassette did not significantly affect the growth characteristics of rNDV-IBV-T/B (*p* > 0.05).

### 3.4. Transmission Electron Microscopy

**Figure 3:**
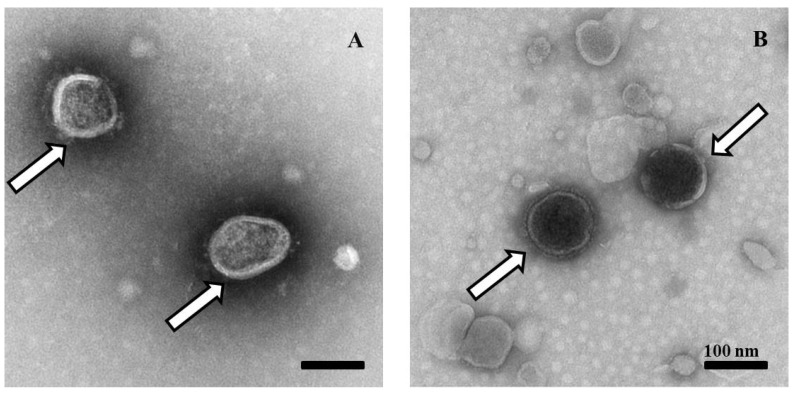
Transmission electron microscopy (TEM) observation on purified rNDV-IBV-T/B and LaSota strains. (**A**) The 20^th^ passage (P20) of rNDV-IBV-T/B, which appears as circles or ovals about 100 to 150 nm in diameter. (**B**) The control parental LaSota virus showed circular particles and the surface of the NDV was characterized by an envelope. Virions are marked by arrows. Scale bar = 100 nm.

To observe the morphology of the recombinant virus rNDV-IBV-T/B, the 20th passage (P20) of rNDV-IBV-T/B and parental LaSota virions were purified by sucrose density gradient centrifugation. Following negative staining, virions were observed by TEM at 98,000× magnification. The rNDV-IBV-T/B virus appeared as circles or ovals about 100 to 150 nm in diameter. The envelope located on the surface layer of the virus can be clearly observed, which is consistent with the morphology of LaSota (Figure 3). TEM indicated that the recombinant virus rNDV-IBV-T/B was successfully rescued and possesses morphology identical to the parental virus.

### 3.5. Hemagglutination Inhibition(HI) and Neutralizing Antibodies Responses 

HI assays were performed according to the standard protocol of OIE and used to assess the level of antibodies mounted against NDV in serum samples of chickens 21 days after immunization. The mean HI titers of NDV in serum samples of chickens immunized with rNDV-IBV-T/B, LaSota, and PBS were 6.6 ± 0.3, 6.7 ± 0.4, and 0, respectively, which are expressed as log2 mean plus standard deviation. There was no significant difference between the rNDV-IBV-T/B and LaSota groups, but HI titers were significantly higher than in the PBS group (*p <* 0.05) (Table 2). This result indicates that rNDV-IBV-T/B could induce high levels of HI titer as LaSota vaccine against NDV challenge.

Virus neutralization (VN) assays were performed according to the standard protocol of OIE. The VN titer of rNDV-IBV-T/B group against IBV M41 strain showed approximately 6.82 ± 0.26, which was significantly higher than the LaSota and PBS groups’ (no VN activity detected) (*p <* 0.05). The VN antibodies induced by rNDV-IBV-T/B administration could effectively neutralize heterologous IBV strain, indicating that the recombinant vaccine could protect against heterologous IBV challenge.

### 3.6. Protective Efficacy of rNDV-IBV-T/B against Velogenic NDV and Virulent IBV Challenge in Chickens

**Figure 4:**
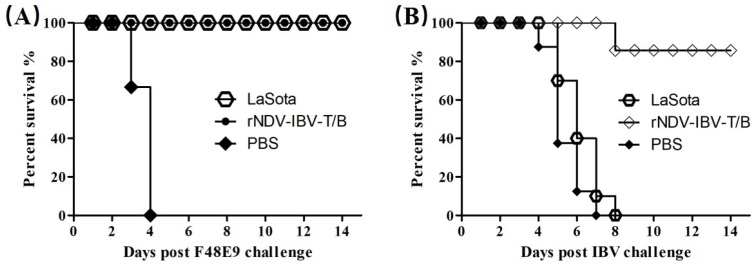
Protective efficacy of vaccines against NDV and IBV challenge. SPF chickens (15 per group) were vaccinated with rNDV-IBV-T/B, LaSota, or PBS. Three weeks post-immunization, chickens were challenged with 10^6^ ELD_50_ of the NDV F48E9 strain (**A**) and IBV M41 strain (**B**). Symptoms and mortality were monitored daily for two weeks, and the survival rate of was calculated and compared by log-rank tests.

To evaluate the efficacy of the multi-epitope vaccine in protecting against IBV and NDV, one-day-old SPF chicks were immunized with rNDV-IBV-T/B virus or PBS via the oculonasal route. At three weeks post-immunization, the chickens were challenged with lethal doses of the NDV F48E9 strain or IBV M41 strain. Chickens inoculated with PBS manifested clinical symptoms on day two post NDV challenge, and all these chickens died within four days. Compared to chickens immunized with parental LaSota and chickens inoculated with PBS, chickens immunized with rNDV-IBV-T/B showed no apparent symptoms post NDV challenge (*p* < 0.05). The rNDV-IBV-T/B immunized group exhibited protective efficacy equivalent to the parental LaSota group (Figure 4A). The clinical symptoms were recorded every day for 14 days post IBV challenge. Most chickens vaccinated with rNDV-IBV-T/B showed significantly less severe clinical signs after IBV challenge, and the survival rate of the rNDV-IBV-T/B group was 90%, with only a single chicken dying on day eight (Figure 4B). In contrast, chickens inoculated with PBS and LaSota vaccine showed severe tracheal rales, gasping and severe respiratory distress; all chickens in these two groups died within eight days post IBV challenge. The protection of chickens vaccinated with the multi-epitope vaccine was significantly higher than that of the PBS and LaSota vaccine groups (*p* < 0.05). The same protection rate was observed in two additional immune-protection tests conducted in parallel. These results confirm that chickens vaccinated with rNDV-IBV-T/B vaccine are substantially protected against NDV and IBV challenge.

### 3.7. Virion Shedding Post-IBV Challenge

**Figure 5:**
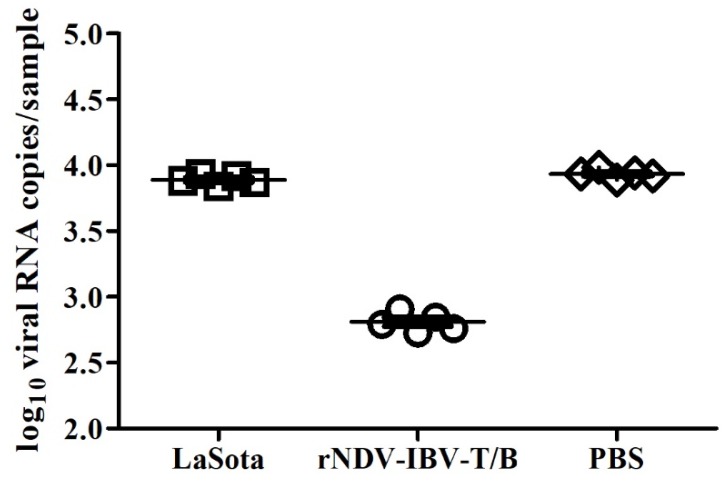
RT-qPCR quantitation of IBV viral shedding in challenged chickens. Four days post-IBV challenge, tracheal swab samples were collected from five chickens and viral RNA was quantitated via RT-qPCR. Copy numbers of IBV viral RNA were determined using a standard curve and log10 values plotted on the scatter plot shown. Horizontal lines indicate means and error bars indicate ± SEM.

Tracheal swab samples taken from five chickens of IBV challenge groups on 4 dpc were analyzed for IBV virions shedding via Taqman probe Real-time qRT-PCR (expressed as means ± SEM). Chickens vaccinated with rNDV-IBV-T/B showed low levels of viral load (647.80 ± 49.65) in the trachea, which was significantly lower than those inoculated with PBS (8591.25 ± 311.10) or LaSota (7742.60 ± 298.50) (*p <* 0.05) (Figure 5). The result demonstrated that rNDV-IBV-T/B vaccination significantly decreases IBV virion shedding as compared with chickens not vaccinated with the IBV epitope-based vaccine (*p* < 0.05). It is probable that rNDV-IBV-T/B effectively activated the immune response and suppressed viral replication to protect chickens against lethal dose of IBV challenge.

### 3.8. Ciliostasis of Vaccinated Chickens

The ciliostasis scores between chickens immunized with rNDV-IBV-T/B, LaSota, and PBS control after a lethal dose of IBV challenge were calculated (Table 3). The maximum mean score of ciliostasis was 39.4 in the PBS control group, and the minimum was 4.2 in the rNDV-IBV-T/B group. The mean ciliostasis score of LaSota was 37.6. This result indicated that rNDV-IBV-T/B reduced the pathogenicity of IBV toward the trachea. Taking the IBV challenge mortality and ciliary activity assay results together, we conclude that rNDV-IBV-T/B induced an effective immune response against the IBV M41 strain.

## 4. Discussion

Infectious bronchitis (IB) and Newcastle disease (ND) are two major infectious diseases of the poultry production industry. Especially in layer and breeder chickens, a decline in egg production alone can cause significant economic losses. However, IBV and NDV live vaccines probably mutually interfere with concurrent immunization [45,46,47]. Furthermore, a live-attenuated IBV vaccine is at risk of virulence recovery, contributing to emergence of new IBV variants. Thus, it is necessary to develop a novel bivalent vaccine protect against NDV and IBV. In recent decades, attempts have been made to develop recombinant vaccines using NDV [48,49,50], adenovirus [51], duck enteritis virus [52], and avian metapneumovirus [53] viral backbones expressing the S1 or S2 portion of the IBV spike gene with differing levels of protection from IBV challenge. Among them, the NDV vector shows great promise due to its excellent characteristics, such as rapid and long-lasting immune response, low production costs, convenience of administration, and genetic stability [45]. The avirulent NDV strain LaSota is an ideal vector that has been approved for use as a vaccine and is widely used worldwide [49]. It should be noted that the maternally derived antibodies of one-day-old chicks probably interfere with LaSota vaccine immunity. Thus, NDV maternal antibodies might be investigated before LaSota vaccination on commercial chickens. 

In previous study, the immune-protection experiment demonstrated that multi epitope-based DNA vaccine *pV-S1B+S1T* could provide effective protection against a lethal dose of homologous IBV SH1208 challenge [26,32]. This result provides a solid foundation to develop the IBV multi-epitope vaccine applied in more vectors. Furthermore, we investigated if the IBV multi-epitope-based vaccine could protect against IBV heterologous challenge. Thus, in this study, we employed NDV LaSota as a backbone vector to express this multi-epitope cassette based on a reverse genetic system and successfully obtained a recombinant NDV candidate vaccine, rNDV-IBV-T/B. The genetic stability of rNDV-IBV-T/B was confirmed by DNA sequencing. The morphology of the recombinant progeny virus remained the same as the parental LaSota strain according to TEM observation. The rNDV-IBV-T/B possessed similar MDT and ICPI index as compared with the parental LaSota (*p* > 0.05). 

The immune-protection experiment showed that a single immunization of one-day-old chicks with rNDV-IBV-T/B could protect 100% and 90% chicks against lethal doses of F48E9 and M41 strain, respectively. The IBV protection rate was analogous to that of a recombinant NDV expressing the full-length S protein of the IBV M41 strain, reported by Shirvani, et al. [48]. These authors found that only recombinant NDV, expressing the full-length S protein, could completely protect against IBV challenge; recombinant NDV expressing only S1 or S2 subunits could not. In contrast, in this study, the rNDV-IBV-T/B, expressing epitopes derived from the S1 subunits of the Holte, Australian T, and QX-like SH1208 IBV strains, provided 90% protection against heterologous IBV M41 strain challenge. We speculate that the conserved epitopes, especially neutralizing epitopes, play an important role in the heterologous IBV cross-protection. [54,55]. Whether the rNDV-IBV-T/B vaccine potentially broadly protects against more heterologous IBV strains needs to be determined in further experiments.

Abozeid et al. [50] reported a similar design strategy as that of Shirvani et al. [48] but modified the IBV S protein and rescued three recombinant LaSota strains. Immune-protection experiments show that candidate vaccines can alleviate the severity of clinical symptoms but does not reduce virus shedding. However, prime-boost immunization with rNDV expressing wild-type IBV S protein can significantly reduce tracheal virus shedding after IBV attack. In contrast, our study found that rNDV-IBV-T/B vaccination strongly protected the chickens, as well as significantly inhibited IBV virus shedding, while also maintaining trachea ciliary activity. This is probably due to the high level of neutralizing antibodies elicited by the IBV multi-epitope vaccine effectively neutralizing the virus. Toro et al. employed the prime-boost immunization strategy to evaluate the efficacy of the recombinant NDV vaccine, rLS/IBV.S2. The results demonstrated that overexposing IBV S2 to the chicken immune system by means of a vectored vaccine, followed by boosting with the whole virus, protects chickens against heterologous IBV [56]. This strategy is a practical immunization procedure applied to protect against various pathogens [57]. This study shows that rNDV-IBV-T/B vaccine could cross-protect against the heterologous IBV M41 strain and indicates that the potency of the rNDV-IBV-T/B could perhaps facilitate the simplified immunization procedures and reduce the production cost. Even more, the results described in this study were obtained with a single-dose immunization, which is highly desirable in poultry vaccines.

## 5. Conclusions

In summary, the findings of this study support that NDV is an outstanding vaccine vector for expressing an IBV multi-epitope protein as a bivalent vaccine against NDV and IBV. Furthermore, the rNDV-IBV-T/B provides an alternative strategy for the development of a cost-effective and extensively immune-protective vaccine for the control of variant IBV infection. 

## Figures and Tables

**Table 1:** Biological assessments between rNDV-IBV-T/B and parental LaSota.

Virus	HA	EID_50_	TCID_50_	MDT (h)	ICPI
LaSota	2^10^	6.5 × 10^8^	3.6 × 10^7^	115	0.10
rNDV-IBV-T/B	2^10^	6.7 × 10^8^	3.7 × 10^7^	118	0.05

Abbreviations: HA, hemagglutination titer; EID_50_, 50% egg infectious dose in embryonated eggs; TCID_50_, 50% tissue infectious dose on DF-1 cells; MDT, mean death time in embryonated eggs; ICPI, intracerebral pathogenicity index in one-day-old chickens.

**Table 2:** Serum NDV- and IBV-specific antibody response of chickens post-immunization.

Treatment	HI Titer	VN Titer
LaSota	6.7 ± 0.41	0
rNDV-IBV-T/B	6.6 ± 0.32	6.82 ± 0.26
PBS	0	0

The hemagglutination inhibition (HI) titer against NDV and virus neutralization (VN) antibody titer against IBV are expressed as mean log2 ± standard deviation.

**Table 3:** Result of ciliostasis test scores and survival of vaccinated chickens challenged with IBV, 3 weeks post-vaccination.

Group	Ciliostasis Score ^a^	Protection Rate ^b^ (%)
LaSota	37.6	0
rNDV-IBV-T/B	4.2	90
PBS	39.4	0

**^a.^** Mean ciliostasis score per chicken for 10 tracheas examined in each group; maximum possible score (no protection) = 40. **^b^** Protection rate in each group based on survival following IBV challenge.
